# Microscale flower-like magnesium oxide for highly efficient photocatalytic degradation of organic dyes in aqueous solution†

**DOI:** 10.1039/c8ra10385b

**Published:** 2019-03-05

**Authors:** Yajun Zheng, Liyun Cao, Gaoxuan Xing, Zongquan Bai, Jianfeng Huang, Zhiping Zhang

**Affiliations:** School of Material Science and Engineering, Shaanxi University of Science and Technology Xi'an 710021 China caoliyun@sust.edu.cn; School of Chemistry and Chemical Engineering, Xi'an Shiyou University Xi'an 710065 China zhangzp0304@gmail.com +86 29 8838 2693 +86 29 8838 2694

## Abstract

Flower-like MgO microparticles with excellent photocatalytic performance in degradation of various organic dyes (*e.g.*, methylene blue, Congo red, thymol blue, bromothymol blue, eriochrome black T, and their mixture) were synthesized by a facile precipitation method *via* the reaction between Mg^2+^ and CO_3_^2−^ at 70 °C. The reaction time was found to be crucial in determining the final morphology of flower-like MgO. After studying the particles from time-dependent experiments, scanning electron microscope observation, Fourier transform infrared spectra and thermogravimetric analyses demonstrated that the formation of flower-like particles involved a complex process, in which agglomerates or rod-like particles with a formula of *x*MgCO_3_·*y*H_2_O (*x* = 0.75–0.77 and *y* = 1.87–1.96) were favorably formed after the initial mixture of the reactants. Owing to the chemical instability, they would turn into flower-like particles, which had a composition of *x*MgCO_3_·*y*Mg(OH)_2_·*z*H_2_O (*x* = 0.84–0.86, *y* = 0.13–0.23, and *z* = 0.77–1.15). After calcination, the generated product not only possessed a superior photocatalytic performance in degradation of organic dyes (100 mg L^−1^) under UV light irradiation in contrast to other morphologies of MgO and other related state-of-the-art photocatalysts (*e.g.*, N-doped TiO_2_, Degussa P25 TiO_2_, ZnO, WO_3_, α-Fe_2_O_3_, BiVO_4_, and g-C_3_N_4_), but also could be used for five cycles, maintaining its efficiency above 92.2%. These capacities made the flower-like MgO a potential candidate for polluted water treatment. Also, the underlying photocatalysis mechanism of MgO was proposed through radical trapping experiments.

## Abstract 2

Flower-like MgO microparticles with excellent photocatalytic performance in degradation of various organic dyes were synthesized by a facile precipitation method *via* the reaction between Mg^2+^ and CO_3_^2−^ at 70 °C.

## Graphical abstract

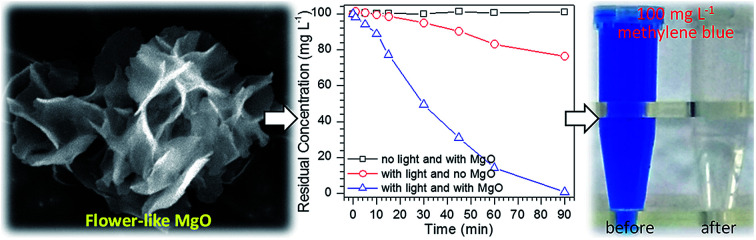

## 1. Introduction

Organic dyes, being discharged from the textile, food, cosmetics, and pharmaceutical industries, are a class of harmful pollutants,^1^ and pose a series of threats to public health and ecological systems because of their toxicity and accumulation. To date, various biological, physical, and chemical protocols have been developed to handle dye-contaminated effluents. Biological methods are environmentally friendly and retain the quality of environments in the remediation process,^2,3^ whereas the related techniques are often costly, ineffective and non-destructive to organic pollutants. Traditional physical techniques such as adsorption have demonstrated superior performance in removal of organic dyes from aqueous solution,^4,5^ but they require an extra and tedious treatment after adsorption for regeneration of used adsorbents. Degradation of organic dyes in industrial effluents through chemical remediation has also received tremendous attention on account of its immense applications. Among the developed methods, photocatalytic degradation using renewable UV/sun light is one of the most promising techniques owing to its simplicity and cost-effectiveness.^6–8^

Numerous metal oxides (*e.g.*, TiO_2_,^9^ ZnO,^10^ Fe_2_O_3_ ^11^ and WO_3_ ^12^), owing to their high chemical stability, nontoxicity, high activity, and low cost, have been extensively used as photocatalytic materials for degradation of organic dyes, and their catalytic mechanisms have also been well documented.^13^ Magnesium oxide (MgO), as a versatile oxide material, has found widespread applications in the fields of adsorption,^5^ catalysis,^14^ ceramics,^15^ antibacterial materials,^16^ paint,^17^ and superconductor products.^18^ Some attempts^19–26^ have also been made to employ MgO as a photocatalyst for degradation of various dyes in aqueous solution based on its large band gap, low dielectric constant and refractive index. For example, Demirci and colleagues^27^ studied the photocatalytic degradation rate of methylene blue solution (6 × 10^−6^ M, 1.9 mg L^−1^) under the catalysis of nanoscale MgO by means of flame spray pyrolysis and sol–gel techniques. Both types of generated MgO particles demonstrated comparable activity for the degradation of methylene blue, and the degradation efficiencies were 94% and 90% after 240 min, respectively. Mantilaka *et al.*^26^ reported that in contrast to MgO nanospheres, electrospun MgO nanofibers demonstrated a more complete degradation efficiency to reactive yellow (10 ppm) after 40 min. Mageshwari *et al.*^20^ investigated the photocatalytic activity of flakes-like MgO nanoparticles synthesized by a reflux condensation method, and found that 99.14% of degradation efficiency could be obtained for 15 mg L^−1^ of methyl orange and methylene blue dyes after an irradiation period of 240 min. Salehifar *et al.*^24^ synthesized MgO nanorods *via* a thermal decomposition route, and observed this material exhibiting appreciable photocatalytic activity, and 90% degradation of methylene blue (25 mg L^−1^) was obtained after 180 min. Kumara *et al.*^23^ reported the catalytic activity of MgO nanoparticles generated from low temperature (400 °C) solution combustion method using urea as fuel, and 98% and 91% of methylene blue and methyl orange with a concentration of 60 mg L^−1^ were degraded after a period of 60 min, respectively. Those studies are crucial to better understanding of the feasibility of using MgO as a photocatalyst for degrading organic dyes. But they are mainly limited in nano-scaled particles, which makes it a challenge for the following recycle and regeneration due to small particle sizes. In addition, the concentration of organic dyes for photocatalytic degradation in the literature is generally in the range of 1.9–60 mg L^−1^, not suitable for wastewater with high concentrations of organic dyes. Therefore, it is necessary to develop larger sized MgO (*e.g.*, micro-sized particles) with high efficiency in degradation of organic dyes.

Over the past decades, different protocols, including chemical vapor deposition,^28^ hydrothermal approach,^29^ sol–gel route^30^ and precipitation,^31^ have been developed for preparing MgO particles. Amongst them, precipitation has received considerable attentions due to its unique features such as facile large-scale production, cost-effectiveness and avoidance of organic reagents. In the previous studies,^14,32–35^ we found that various morphologies of micro-sized MgO could be synthesized by precipitation. Inspired by those investigation, herein we developed a micro-sized flower-like MgO, and explored its shape and composition evolution with reaction time. The photocatalytic performance of the as-synthesized MgO was evaluated by monitoring the photodegradation of various organic dyes (*e.g.*, methylene blue, Congo red, thymol blue, bromothymol blue, eriochrome black T, and their mixture) under UV light irradiation. In contrast to other available micro-sized MgO with different morphologies (*e.g.*, nest-like, spherical, rod-like and trapezoidal products), the as-prepared flower-like material illustrated a superior performance, and 100 mg L^−1^ of organic dyes such as methylene blue in aqueous solution could be completely degraded in 90 min. Several techniques, including photoluminescence spectroscopy, UV diffused reflectance spectroscopy, N_2_ physical adsorption and UV-visible spectrometer, were also used to explore the differences between the surface properties of various morphologies of MgO in detail. The underlying photocatalysis mechanism of MgO was also proposed.

## 2. Experimental

### 2.1. Synthesis of flower-like MgO

All reagents (*e.g.*, Mg(NO_3_)_2_·6H_2_O and Na_2_CO_3_) were of analytical grade or better and were used as received without further purification. The procedure was similar to our previous report^14^ but had a different stirring condition. Specifically, 0.04 mol of Mg(NO_3_)_2_·6H_2_O (Guangdong Guanghua Sci-Tech Co., Ltd., Shantou, China) was dissolved into 50 mL of deionized water, followed by transferring into a 250 mL three-necked flask and heated to 70 °C. Under the vigorous stirring (*ca.* 1000 rpm), 100 mL of 0.4 M Na_2_CO_3_ (Tianjin Tianli Chemical Reagents Co., Ltd., Tianjin, China) solution was poured into the Mg(NO_3_)_2_ solution in 10 s, which was then stirred for 3 min. Afterwards, the mixture was maintained at 70 °C for 1 h under static conditions, and the obtained product was collected, filtered off, and washed with deionized water for three times (each with around 110 mL and a washing time of about 50 seconds) followed by 20 mL of absolute ethanol. The flower-like MgO sample was fabricated by calcining the obtained product in air from room temperature to 550 °C in a muffle furnace, and then maintaining at that temperature for 3 h.

### 2.2. Catalyst characterization

The morphology and size of the obtained particles were examined by a JEOL JSM-6390A scanning electron microscope (SEM). The crystal structures of the as-synthesized products were characterized by X-ray diffraction (XRD) on a XRD-6000 diffractometer using Cu Kα radiation. The operation voltage was 40 kV, and the current was 30 mA. The microstructure and crystalline structure of flower-like MgO particles was analyzed by transmission electron microscope (TEM, JEOL JEM-2100 Plus) with an acceleration voltage of 200 kV. Optical band gaps of MgO particles with different morphologies were estimated from UV diffused reflectance spectroscopy (DRS) using a UV-2600 UV-vis spectrophotometer. The optical band gap of MgO particles was calculated according to the previously reported method.^20^ Photoluminescence (PL) spectra were recorded using a Thermo Scientific Lumina spectrofluorometer equipped with 150 W xenon lamp (excitation wavelength = 325 nm). The thermal decomposition behaviors of the MgO precursors were detected by a thermogravimetric analyzer (TGA, Mettler Toledo, TGA/DSC1 STAR System, Switzerland), which was carried out in dynamic nitrogen gas with a heating rate of 10 °C min^−1^. The N_2_ adsorption–desorption isotherm was determined on a Micromeritics ASAP 2020HD88 physisorption apparatus at the temperature of liquid nitrogen, in which the samples were degassed at 350 °C for 4 h before measurement. The surface area was calculated by the Brunauer–Emmett–Teller (BET) method. The concentration of methylene blue in aqueous solutions was measured on a Shimadzu UV-2600 UV-visible spectrometer.

### 2.3. Photocatalytic activity

The photocatalytic performance of the as-prepared MgO particles, N-doped TiO_2_ and ZnO (Hangzhou Zhitai Purification Technology Co., Ltd., Hangzhou, China), WO_3_ (Shanghai Yuanye Bio-Technology Co., Ltd., Shanghai, China), α-Fe_2_O_3_ (Shanghai Yi'en Chemical Technology Co., Ltd., Shanghai, China), BiVO_4_ (Sa'en Chemical Technology Co., Ltd., Shanghai, China), g-C_3_N_4_ (Jiangsu XFNANO Materials Tech Co., Ltd., Nanjing, China), and Degussa P25 TiO_2_ nanoparticles (Shanghai Bestmore Industry Co. Ltd., Shanghai, China) was evaluated by monitoring the photodegradation of organic dyes such as methylene blue under UV light irradiation with a mercury lamp (power: 400 W, wavelength: 365 nm). In a typical process, 10 mg of MgO particles were added into 20 mL of methylene blue solution with a concentration of 100 mg L^−1^. The distance between the lamp and the suspension containing MgO particles was 15 cm. After the mixture was irradiated with UV radiation under gently magnetic stirring for 90 min, the generated suspension was taken and centrifuged (8000 rpm for 6 min) to separate the reaction solution and the MgO photocatalyst. Then the supernatant was analyzed by a Shimadzu UV-2600 UV-visible spectrometer. For the recycle and regeneration of flower-like MgO particles, the collected catalyst was first heated to 400 °C and then maintained at that temperature for 3 h in a nitrogen atmosphere followed by calcination at 550 °C for 3 h in air following our recent study.^14^

The degradation efficiency of studied dye on MgO was evaluated using eqn (1),^36^ and the photocatalytic kinetic was characterized by eqn (2).^20^1
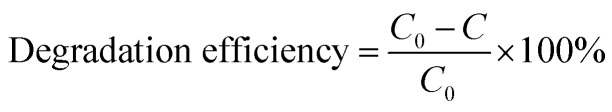
2
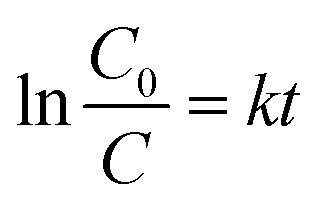
where *C*_0_ is the initial concentration of dye solution, *C* is the concentration of dye solution after irradiation in a fixed interval, *k* is the degradation rate constant, and *t* is the reaction time (min).

## 3. Results and discussion

### 3.1. Synthesis and characterization of flower-like MgO catalyst

**Fig. 1:**
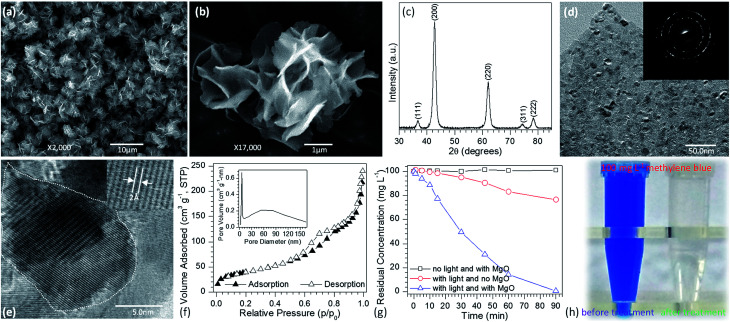
Representative SEM images of flower-like MgO particles generated by calcining its precursor which was synthesized by reaction between Mg(NO_3_)_2_ and Na_2_CO_3_ under a temperature of 70 °C: (a) panoramic morphology and (b) individual particle; (c) XRD pattern; (d) and (e) HRTEM images of flower-like MgO [the right upper insets in (d) and (e) are, respectively, their corresponding selected area electron diffraction (SAED) pattern and magnified area of the region of white dot curve]; (f) N_2_ adsorption–desorption isotherms and pore size distribution (inset) of the generated product; (g) photocatalytic degradation of methylene blue by the as-prepared MgO under different conditions as marked; (h) comparison of the color change of 100 mg L^−1^ methylene blue aqueous solution before and after treatment with the developed MgO.

The flower-like MgO was synthesized by the reaction between 0.04 M of Mg(NO_3_)_2_ and Na_2_CO_3_ without addition of any organic or inorganic additives at 70 °C. After mixing, the reaction solution was stirred for 3.0 min and then maintained under static conditions for 1 h. Fig. 1a and b shows the representative SEM images of the as-prepared flower-like MgO particles by calcining their precursors at 550 °C for 3 h. Fig. 1a presents the lower-magnification SEM image of the synthesized particles. Obviously, the panoramic morphology of the sample consists of flower-like particles with diameters of 3.8–6.0 μm. Fig. 1b shows a magnified view of an individual particle, and careful observation could be found that the particle is composed of sheet-like structure with a thickness of 16–20 nm. The purity and crystallinity of the as-synthesized product were examined by using powder XRD (Fig. 1c). All the diffraction peaks could be indexed to a cubic structure of MgO with a crystallite size of 7.2 nm (JCPDS no. 45-0946). No other peaks were observed, indicating the high purify of the obtained particles. HRTEM images demonstrate that the flower-like MgO structure is polycrystalline in nature (Fig. 1d) and is made of domains of single crystalline nanoparticles and amorphous particles (Fig. 1e). These characteristics suggest that the developed MgO particles have the feature of mesocrystal material, in good agreement with our recent study on parallelogrammic MgO.^34^ N_2_ adsorption–desorption measurement was also carried out to study the surface structure and porosity of generated MgO. As shown in Fig. 1f, a type-IV characteristics with a significant H3 loop^37^ was observed for N_2_ adsorption–desorption isotherms. The BJH pore size distribution (inset in Fig. 1f) obtained from the isotherm indicated that the obtained flower-like MgO possessed hierarchical pore structure including mesopores and macropores. Their pore sizes were centered around 5.2 nm and 53.5 nm, respectively, in consistance with the H3 loop in the N_2_ adsorption–desorption isotherms. From those measurement, the obtained flower-like MgO had a BET specific surface area, pore volume, and average pore diameter of 142.9 m^2^ g^−1^, 0.37 cm^3^ g^−1^ and 8.4 nm (Table 1), respectively.

**Table 1:** Texture properties of various morphologies of MgO particles

Morphology	Specific surface area^a^ (m^2^ g^−1^)	Average pore diameter^b^ (nm)	Pore volume (cm^3^ g^−1^)	Crystallite size^c^ (nm)	Band gap energy^d^ (eV)
Flower-like	142.9	8.4	0.37	7.2	5.23
Nest-like	141.8	11.3	0.49	8.1	5.11
Spherical	136.1	12.3	0.58	8.2	5.01
Rod-like	137.8	5.9	0.31	7.5	5.15
Trapezoidal	31.8	19.5	0.13	23.1	4.99

a Using the standard Brunauer–Emmett–Teller (BET) method.

b Using the Barrett–Joyner–Halenda (BJH) method.

c Using the Debye–Scherrer formula based on the full width at half-maximum (fwhm) of the (200) plane.

d Estimated from UV diffused reflectance spectroscopy (DRS).

**Fig. 2:**
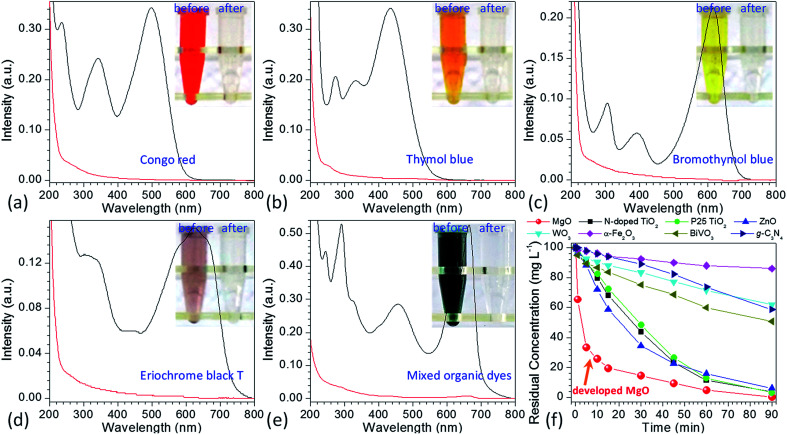
UV-vis spectra of initial (a) Congo red (100 mg L^−1^), (b) thymol blue (100 mg L^−1^), (c) bromothymol blue (100 mg L^−1^), (d) eriochrome black T (100 mg L^−1^), (e) their mixed solution plus methylene blue (the concentration of each dye was 100 mg L^−1^) being treated with 10 mg of the as-prepared MgO particles (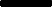 means the solution before treatment, and 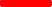 means the solution after treatment; irradiation time: 90 min; solution volume: 20 mL; insets are the photographic images of the corresponding solutions after 0 min and 90 min), (f) comparison of the kinetic curves for the degradation of 100 mg L^−1^ methylene blue between developed MgO particles and the available state-of-the-art photocatalysts as indicated in this figure.

To demonstrate the performance of obtained flower-like MgO in the treatment of polluted water, methylene blue was selected as a model organic pollutant. UV-vis absorption spectroscopy was employed to evaluate the adsorption behavior of the aqueous solution after treatment. The characteristic absorption peak of methylene blue at 664 nm was used to monitor the removal efficiency of flower-like MgO. To exclude the influences of MgO adsorption and UV irradiation on the removal, the variation in the concentration of 100 mg L^−1^ of methylene blue aqueous solution under different experimental conditions was studied in detail as shown in Fig. 1g and S1 (see the ESI†). For the reaction system without irradiation but with MgO, the concentration of methylene blue after 90 min of stirring kept constant, suggesting that the flower-like MgO had very weak adsorption ability under the current condition. As the solution was exposed under the UV irradiation but without MgO, it could be found that 23.6% of methylene blue were photodegraded from the aqueous solution after an irradiation period of 90 min, and the remaining concentration was 76.4 mg L^−1^. When the as-prepared MgO particles were involved in the reaction system, the concentration of methylene blue gradually decreased with extension of irradiation time, and 99.8% of degradation efficiency was obtained after 90 min. Fig. 1h compares the color change in 100 mg L^−1^ of methylene blue solution before and after treatment with the flower-like MgO under UV irradiation. It is apparent that the as-synthesized MgO is a highly efficient catalyst for the degradation of methylene blue in aqueous solution. We also found that it was an excellent catalyst in degradation of other dyes such as Congo red, thymol blue, bromothymol blue, and eriochrome black T. Fig. 2a–d shows that when 100 mg L^−1^ of the above dye solution was treated with the as-synthesized MgO, respectively, the degradation efficiency could be more than 99.9%. In the real wastewater, dye generally exits as a mixture rather than a single one. To explore the capacity of the developed MgO, we treated the mixture solution containing the above dyes as well as methylene blue, and their concentrations were all 100 mg L^−1^. It is noticeable that using the flower-like particles, almost all dyes could be degraded in 90 min, and its efficiency was beyond than 99% (Fig. 2e). We also observed the performance of the flower-like MgO was superior to other related state-of-the-art photocatalysts in the literature (*e.g.*, N-doped TiO_2_,^38^ Degussa P25 TiO_2_,^39–41^ ZnO,^42^ WO_3_,^43^ α-Fe_2_O_3_,^44^ BiVO_4_,^45^ and g-C_3_N_4_ ^46^) in degradation of organic dyes under comparable conditions (Fig. 2e, f and S2–S4 in the ESI†). For example, with the developed MgO as catalyst, more than 80% of methylene blue (100 mg L^−1^) could be degraded in 15 min, whereas less than 30% was degraded using P25 TiO_2_.^39–41^ With the extension of illustration time up to 90 min, the degradation efficiency was more than 99% using the flower-like MgO, and the value for P25 TiO_2_ was around 96% (Fig. 2f, S2 and S3†). Moreover, the performance of the flower-like MgO (Fig. 2e) was much better than the available most used photocatalysts (Fig. S4†) in treatment of the mixed organic dyes containing methylene blue, Congo red, thymol blue, bromothymol blue, and eriochrome black T with each concentration of 100 mg L^−1^. These results illustrate that the currently developed MgO is a promising catalyst in photodegradation of organic dyes in aqueous solution.

### 3.2. Evolution in the morphology and composition of flower-like MgO precursor

**Fig. 3:**
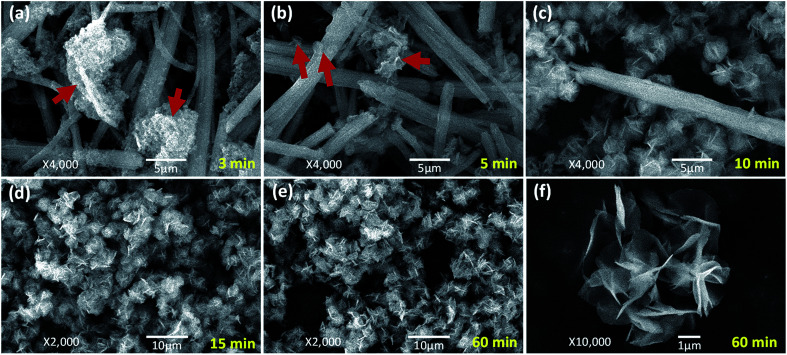
Typical SEM images of the obtained particles from the reaction between Mg(NO_3_)_2_ and Na_2_CO_3_ under a temperature of 70 °C after different reaction periods: (a) 3 min, (b) 5 min, (c) 10 min, (d) 15 min, (e) and (f) 60 min.

To well control the shape and structure of the flower-like MgO, it is crucial to get a better understanding on their formation and evolution. Fig. 3 illustrates the typical SEM images of the particles synthesized from various reaction times at 70 °C. It is evident that after stopping stirring the reaction solution containing Mg(NO_3_)_2_ and Na_2_CO_3_ under 70 °C (3 min, Fig. 3a), the primary particles tended to form agglomerates built by the fine grains with sizes of 90–100 nm and rod-like particles with diameters of 0.2–2.1 μm. As the reaction period was increased to 5 min (Fig. 3b), rod-like particles were obtained, and there were some flower-like particles involved in the product. More interesting was that there were some sheet-like particles incubating at the surface of generated rod-like particles as indicated in red arrow, which could be the primary unit for flower-like particles. Further increasing the reaction time to 10 min led to the gradual disappearance of rod-like particles, and promoted the generation of flower-like particles. From Fig. 3c, it is apparent that there are few rod-like particles in the sample, and most of them are flower-like particles. With extension of the reaction period up to 15–60 min (Fig. 3d–f), only flower-like particles were produced although there were some tiny differences between their surface structures, presumably due to their different compositions as discussed below. From the above, it could be envisioned that the formation of flower-like particles experienced a series of complex processes, in which agglomerates were first formed after mixing Mg(NO_3_)_2_ and Na_2_CO_3_. Due to the thermal instability,^33^ agglomerates began to transfer into rod-like particles, and the latter further became into flower-like particles with extension of reaction time at 70 °C.

**Fig. 4:**
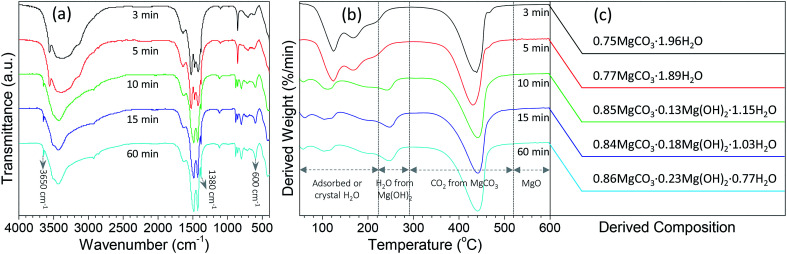
(a) FT-IR spectra and (b) derivative thermogravimetric (DTG) curves of the particles from the reaction between Mg(NO_3_)_2_ and Na_2_CO_3_ under a temperature of 70 °C after various reaction periods, and (c) their derived compositions from thermogravimetric data.

In order to get a better understanding on the shape evolution of flower-like particles, the structures and compositions of the samples from different reaction times (3, 5, 10, 15 and 60 min) were characterized by FT-IR and TG-DTG analyses. Fig. 4a shows the typical IR spectra of these particles from various times. It is apparent that the reaction time had a pronounced effect on the composition of the resulting products. As the reaction periods were 3 min and 5 min, both IR spectra were very similar to those of MgCO_3_·3H_2_O^47^ with the CO_3_^2−^ adsorption bands of 854 cm^−1^ (υ_2_ mode), 1100 cm^−1^ (υ_1_ mode), 1430, 1480 and 1520 (υ_3_ mode) as well as the O–H bending mode of water at 1650 cm^−1^.^32^ In the range of 2800–3700 cm^−1^, there is a broad band resulting from different numbers of water of crystallization, and the band at 3560 cm^−1^ could be attributed to the O–H stretching vibration of water. When the reaction period was extended to more than 10 min, the IR spectra of generated products had much differences from those generated from reaction periods of 3 min and 5 min. The CO_3_^2−^ bending vibrations split into three bands at 802, 854, and 883 cm^−1^, and the triple adsorption bands ranging from 1400 to 1550 cm^−1^ became only two at 1430 and 1490 cm^−1^. In addition, the bands between 3600 and 3200 cm^−1^ became narrower, and a sharp band corresponding to the free O–H vibration occurred at 3650 cm^−1^. According to the previous reports,^32,33,48,49^ the above adsorption bands suggested that the products from a reaction time of more than 10 min possessed the features of Mg_5_(CO_3_)_4_(OH)_2_·4H_2_O. Careful observation could be also seen that with variation in the reaction time from 10 to 60 min, the bands at 3650, 1380, and 600 cm^−1^ demonstrated an increasing trend, revealing that there were some differences between those structures. To figure out the difference between them, the weight loss in the derivative thermogravimetric (DTG) curves of the samples from different reaction times was divided into different temperature stages [50–290 °C due to adsorbed or crystal H_2_O, 223–290 °C due to H_2_O from decomposition of Mg(OH)_2_ if there was a peak around 247 °C, 290–520 °C due to CO_2_ from decomposition of MgCO_3_, and above 520 °C due to the formation of MgO] as shown in Fig. 4b. After a calculation from the weight loss in various stages, it is clear that the composition of the samples from different reaction times varied significantly (Fig. 4c). For example, as the reaction time was 3 min, the weight loss in the range of 50–290 °C was around 35.3%, the weight loss from 290 to 600 °C was 34.4%, and above 600 °C the remaining weight was about 30.3%. After a rough calculation by dividing their corresponding molecular weights of H_2_O (18.01), CO_2_ (44.01) and MgO (40.30), the product had a simple formula of 0.75MgCO_3_·1.96H_2_O. With extension of the reaction time to 5 min, the composition of resulting product (0.77MgCO_3_·1.89H_2_O) was very similar to that from 3 min, which to a certain degree explained why both IR spectra were close (Fig. 4a). When the reaction time was more than 10 min, the formula of generated samples had an analogous composition but were significantly different from the products from 3 and 5 min. Moreover, the amount of Mg(OH)_2_ demonstrated a gradual increasing trend, whereas an opposite tendency was observed for their water contents, ranging from 0.85MgCO_3_·0.13Mg(OH)_2_·1.15H_2_O to 0.86MgCO_3_·0.23Mg(OH)_2_·0.77H_2_O. These results suggest that the reaction time was a crucial factor in determining the shapes and compositions of obtained products after mixing Mg(NO_3_)_2_ and Na_2_CO_3_ at 70 °C. Namely, after the initial mixture of reactants, the product tended to become into agglomerates or rod-like particles (Fig. 3a and b) with a simple formula of *x*MgCO_3_·*y*H_2_O (*x* = 0.75–0.77 and *y* = 1.87–1.96). Owing to the chemical instability, they would turn into flower-like particles with a composition of *x*MgCO_3_·*y*Mg(OH)_2_·*z*H_2_O (*x* = 0.84–0.86, *y* = 0.13–0.23, and *z* = 0.77–1.15).

### 3.3. Comparison between the performance of flower-like MgO and other morphologies of MgO in photocatalytic degradation of methylene blue

**Fig. 5:**
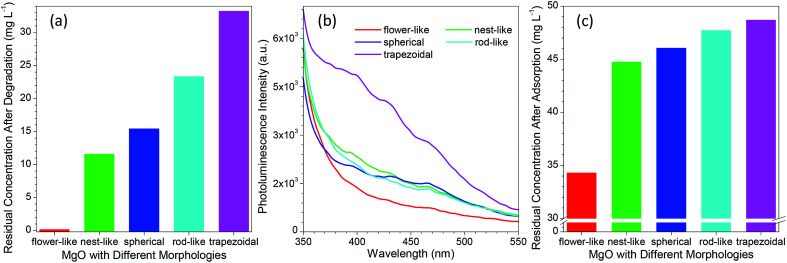
(a) Comparison of the residual concentration of 100 mg L^−1^ methylene blue aqueous solution after treatment with different morphologies of MgO particles; (b) comparison of the photoluminescence spectra of MgO particles with different morphologies; (c) comparison of the residual concentration of 50 mg L^−1^ methylene blue aqueous solution after adsorption with different morphologies of 10 mg MgO particles for 90 min without any UV irradiation.

To date, various morphologies of micro-sized MgO have been developed *via* different protocols.^31–35,50–54^ As reported in many studies,^14,29,34,55–59^ the catalytic performance of MgO was highly dependent on its morphology. To get an insight into the difference between the developed flower-like MgO and others in photocatalytic degradation of methylene blue, several morphologies (*e.g.*, nest-like, spherical, rod-like and trapezoidal) of MgO were prepared following the previous reports.^32–34^ Their performance was evaluated by the residual concentration of 100 mg L^−1^ methylene blue after photocatalytic degradation for 90 min. As shown in Fig. 5a and S5a (see the ESI†), the catalytic performance of MgO significantly changed with variation in the morphology, in good agreement with the previous reports.^14,29,34,55–59^ Among them, flower-like MgO demonstrated a superior performance to others in degradation of methylene blue, and trapezoidal MgO gave the poorest capacity. The activity of a catalyst is generally related to their physicochemical properties such as specific surface, average pore, pore volume and crystallite size, but no direct correlation was found between them and the photocatalytic performance of various morphologies of MgO (Table 1 and Fig. 5a). In addition, little relationship was found between the performance of those MgO particles and the optical band gap energy (as listed in Table 1) estimated from UV DRS (Fig. S6 in the ESI†).^20,60^

Although MgO is a typical wide band gap insulator, its PL property was generally studied due to the presence of various structural defects (*e.g.*, oxygen vacancies, F-centers or F^+^-centers).^61–63^ According to the report by Mageshwari *et al.*,^20^ a higher abundance of PL emission peaks from MgO would lead to a more favorable photocatalytic performance. However, an opposite trend was observed for the MgO with various morphologies in the current investigation despite of the weak PL signal. As shown in Fig. 5b, with varying the MgO from flower-like to trapezoidal structures, the peak intensity of the PL emission in the range of 350–550 nm exhibited a gradual increasing trend although the cases for nest-like, spherical and rod-like MgO were close. A higher intensity in the PL emission, a poor performance in photocatalytic degradation of methylene blue (Fig. 5a). In our opinion, this fact could be ascribed to the different MgO sizes between the previous report (nano-sized particles)^20^ and the current study (micro-sized particles).

We also observed that the adsorption ability of MgO was responsible to the photocatalytic degradation of methylene blue in aqueous solution. Fig. 5c and S5b (see the ESI†) compared the residual concentration of 50 mg L^−1^ methylene blue after adsorption with different morphologies of MgO particles (10 mg) for 90 min without UV light irradiation. It is evident that the developed flower-like MgO possessed the strongest adsorption ability to methylene blue, followed by nest-like, spherical, rod-like, and trapezoidal MgO. This order is in good agreement with their photocatalytic performance (Fig. 5a). This phenomenon reveals that in the photocatalytic degradation process, the adsorption of methylene blue onto the surface of MgO plays a crucial role in determining the final catalytic performance. Despite of the less surface defects of the flower-like MgO relative to others (Fig. 5b), its stronger adsorption ability would make more methylene blue cover at the MgO surface. Under the UV irradiation, methylene blue would more favorably interact with the flower-like MgO, thus leading to its higher efficiency in catalytic degradation.

### 3.4. Photocatalytic activity of flower-like MgO

**Fig. 6:**
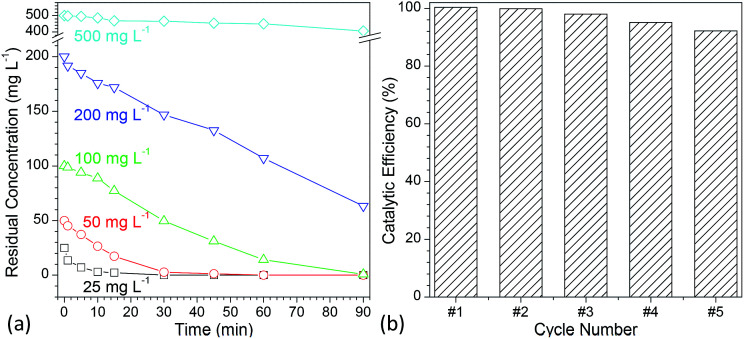
(a) Comparison of the photocatalytic efficiency of flower-like MgO with variation in the concentration of methylene blue aqueous solution ranging from 25 to 500 mg L^−1^ under different irradiation times (0–90 min); (b) reuse of flower-like MgO particles for photocatalytic degradation of methylene blue (concentration of methylene blue: 100 mg L^−1^, irradiation time: 90 min).

To evaluate the photocatalytic activity of the developed flower-like MgO, various concentrations of methylene blue aqueous solutions (25, 50, 100, 200 and 500 mg L^−1^) were employed. Fig. 6a shows the degradation kinetic curves of methylene blue with variation in the reaction time ranging from 0 to 90 min (Fig. S7 in the ESI†). It is apparent that when the concentration was below than 100 mg L^−1^, almost all of methylene blue (>99%) could be completely degraded in 90 min, and the half-life times (*t*_1/2_) for them were 3.47, 6.92 and 24.24 min, respectively (Table 2). When the concentration was increased to 200 mg L^−1^, the catalytic activity of flower-like MgO was sharply decreased, and only 68.35% of methylene blue could be degraded after 90 min. Further increasing the concentration of methylene blue up to 500 mg L^−1^ led to a slower degradation rate, and 18.74% of degradation efficiency was obtained. Its half-life time was as high as 315.07 min. From the above discussion, it could be seen that the developed flower-like MgO was an efficient catalyst in degradation of methylene blue with a concentration below than 100 mg L^−1^, and an increase in the concentration would result in the deterioration of its photocatalytic activity. This could be presumably ascribed to the strong adsorption performance of the flower-like MgO to methylene blue (Fig. 5c), and the active sites at its surface were covered by overloaded dye molecules, thus leading to a decrease in its catalytic activity.

**Table 2:** Kinetic parameters of flower-like MgO for methylene blue degradation

Concentration (mg L^−1^)	*K*_f_ (min^−1^)	*t*_1/2_ (min)	*R* ^2^	Degradation efficiency (%)
25	0.2000	3.47	0.9639	100.00
50	0.1001	6.92	0.9465	100.00
100	0.0286	24.24	0.9501	99.83
200	0.0115	60.27	0.9670	68.35
500	0.0022	315.07	0.9368	18.74

In the development of a catalyst, reusability is another important consideration. Although much effort^19–26^ has been made to use MgO in photocatalytic degradation of various dyes, its reusability is rarely considered. To characterize the reuse of the developed flower-like MgO particles, 100 mg L^−1^ methylene blue was degraded with recycled catalyst prepared following a simple centrifugation and calcination procedure.^14^ As shown in Fig. 6b and S8 (see the ESI†), after five cycles, a degradation efficiency as high as 92.2% could still be obtained. In the previous studies,^14,64–71^ although different shapes of micrometer-sized flower-like MgO have also been reported, few^66,68,69^ are analogous to the shape of obtained MgO in the current study. Also, their performance had much difference. As reported by Cui *et al.*,^68^ the hierarchical flower-like MgO prepared by their method as a catalyst was fragile, and the petals were prone to break into pieces during stirring. This made their catalytic performance drop sharply after a couple of runs. However, the currently developed flower-like MgO still maintained its capacity after five cycles as described above. We also found that the performance of as-prepared flower-like MgO was much superior to the flower-like particles^14^ prepared in a comparable condition but with a low stirring speed (800 rpm, Fig. S9 in the ESI†) although the latter was also composed of nano-sized sheet-like structure and was much more regular in shape than the former. The difference was presumably due to the fact that the currently higher stirring speed (1000 rpm) disturbed the self-assembly behavior of layer-like units into uniform particles, thus leading to a more random sheet-like structure and a higher surface area (142.9 m^2^ g^−1^) than that in the previous study (116.2 m^2^ g^−1^)^14^ despite of comparable crystal structure (Fig. S10†). This promoted a more favorable interaction between the MgO surface and organic dyes in the UV irradiation, and a higher photocatalytic capacity was obtained. Those facts suggest that the currently developed flower-like MgO is a robust photocatalyst in degradation of methylene blue and other organic dyes in aqueous solution.

### 3.5. Possible photocatalytic reaction mechanism

**Fig. 7:**
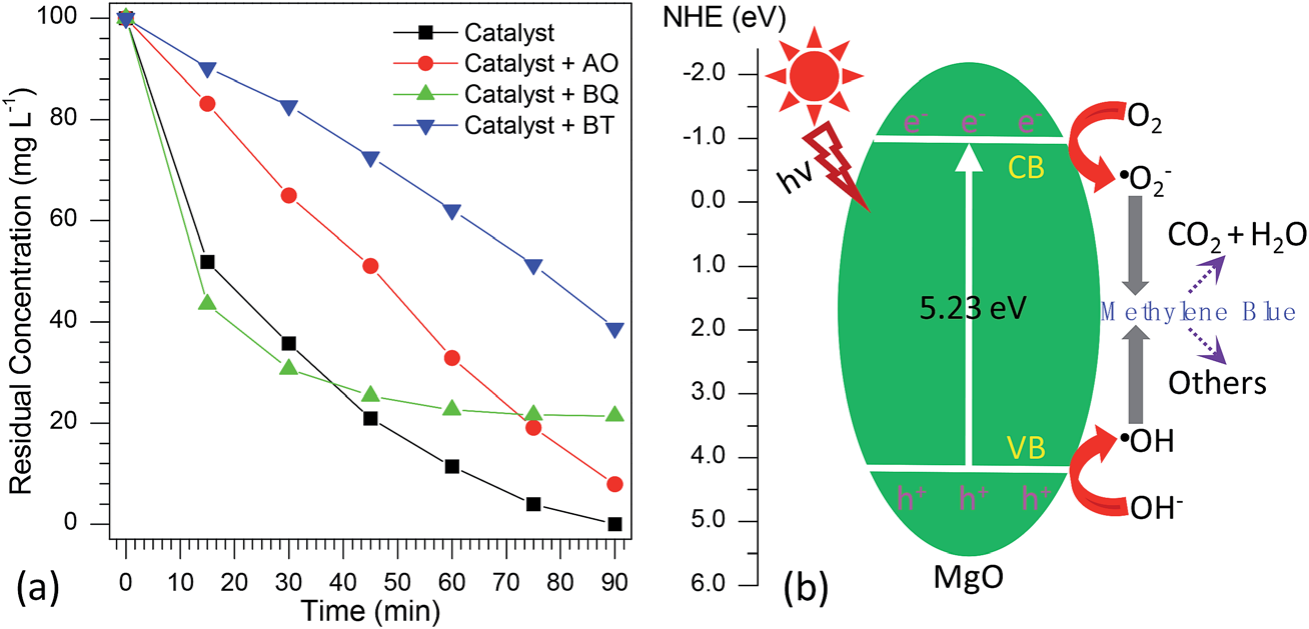
(a) Effect of different scavengers on the photocatalytic degradation percentage of methylene blue over the developed MgO catalyst (AO means ammonium oxalate, BT means *t*-butanol, and BQ means 1,4-benzoquinone); (b) proposed photocatalytic mechanism for flower-like MgO under UV light irradiation.

According to the previous reports,^72–77^ the photocatalytic degradation of organic pollutants occurs mainly through the oxidizability of photogenerated holes (h^+^), hydroxyl radicals (·OH), and superoxide radicals (·O_2_^−^). For the purpose of better understanding the photocatalytic mechanism of methylene blue over flower-like MgO, the trapping experiments of active species involved in the photocatalytic reaction were investigated. In this study, ammonium oxalate (AO, 5.0 mmol L^−1^), *t*-butanol (BT, 5.0 mmol L^−1^) and 1,4-benzoquinone (BQ, 0.1 mmol L^−1^) acting as the scavengers for holes (h^+^), hydroxyl radicals (·OH) and superoxide radicals (·O_2_^−^) were employed. The influence of different scavengers on the degradation efficiency of flower-like MgO is shown in Fig. 7a. It is obvious that in the presence of BQ, it had less effect on the degradation of methylene blue in the reaction period ranging from 0 to 30 min, and after that (30–90 min) the catalytic reaction had been greatly restricted, suggesting that ·O_2_^−^ active species had a pronounced effect on the photocatalytic performance of developed MgO as the reaction proceeded for a long period. However, the photocatalytic activity was gradually decreased by adding AO and strongly suppressed by introducing BT. This fact suggests that both h^+^ and ·OH active species played crucial roles in the removal of methylene blue upon the catalysis of flower-like MgO. Based on the above results, it could be concluded that the effect of active species on photocatalytic activities is as follows: ·OH > h^+^ > ·O_2_^−^.

From the above information, a possible mechanism for the degradation of methylene blue over flower-like MgO is proposed as Fig. 7b. First, the developed MgO was excited upon the effect of UV light illustration. The electrons on the conduction band (CB) of MgO with strong reduction ability could drive the generation of superoxide radical anions (·O_2_^−^) through reducing the adsorbed O_2_ on its surface, whereas the h^+^ on the valence band (VB) of MgO with high oxidation potentials could produce more reactive ·OH radicals for the photodegradation of methylene blue. As illustrated in the radical trapping experiments, the ·OH and h^+^ were the dominant species for attacking methylene blue adsorbed at the surface of MgO into small molecules in the initial stage. In contrast, ·O_2_^−^ and ·OH played more important roles than h^+^ in determining the degradation efficiency of methylene blue with further increase in the reaction period. According to the general view,^78^ photo-induced electrons could not be efficiently produced by MgO with a broad band gap (5.23 eV, Table 1). To our knowledge, in the photo-catalysis process, the photo-induced electrons could rapidly transfer presumably due to the presence of heterojunction in the mesocrystal flower-like MgO (Fig. 1d and e), which might promote the separation of photo-induced electrons and holes and suppress efficiently their recombination. The lower PL intensity of flower-like MgO relative to other morphologies of products (Fig. 5b) also indicated the lower recombination rate of holes and electrons.^75,79,80^ Therefore, flower-like MgO demonstrated excellent photocatalytic activity in degradation of methylene blue to CO_2_, H_2_O and others under UV light illustration.

## 4. Conclusions

In summary, micro-sized flower-like MgO particles with excellent performance in photocatalytic degradation of various organic dyes (*e.g.*, methylene blue, Congo red, thymol blue, bromothymol blue, eriochrome black T, and their mixture) had been successfully prepared by a facile precipitation *via* the reaction between Mg^2+^ and CO_3_^2−^ under a temperature of 70 °C. Time-dependent experiments demonstrated that their formation involved a complex process, in which after the initial mixture of reactants, the product was prone to turn into agglomerates or rod-like particles with a formula of *x*MgCO_3_·*y*H_2_O (*x* = 0.75–0.77 and *y* = 1.87–1.96). Because of the chemical instability, they would transfer into flower-like particles with a composition of *x*MgCO_3_·*y*Mg(OH)_2_·*z*H_2_O (*x* = 0.84–0.86, *y* = 0.13–0.23, and *z* = 0.77–1.15). With extension of the reaction time, the content of Mg(OH)_2_ in the product demonstrated an increasing trend, whereas an opposite tendency was observed for the level of H_2_O. In addition, we found that the performance of the flower-like particles in photocatalytic degradation of organic dyes was superior to other morphologies of MgO (*e.g.*, nest-like, spherical, rod-like and trapezoidal structures) due to the stronger adsorption ability to organic dyes. The developed catalyst also demonstrated an excellent reusability, and after five cycles the degradation efficiency could still be maintained beyond 92%. The superior photocatalytic performance on the degradation of organic dyes suggested that the flower-like MgO was a promising candidate for environmental remediation.

## Conflicts of interest

There are no conflicts to declare.

## Supplementary Material

RA-009-C8RA10385B-s001
