# Supplementary material for: Microscale flower-like magnesium oxide for highly efficient photocatalytic degradation of organic dyes in aqueous solution
Source: RSC Adv. 2019 Mar 5;9(13):7338–48. doi: 10.1039/c8ra10385b (PMC9061167; doi:10.1039/c8ra10385b)
Supplement: RA-009-C8RA10385B-s001 [file RA-009-C8RA10385B-s001.pdf]

Electronic Supplementary Material (ESI) for RSC Advances.
This journal is © The Royal Society of Chemistry 2019

Electronic Supplementary Information

Microscale flower-like magnesium oxide for highly efficient photocatalytic degradation of organic dyes in aqueous solution

*Yajun Zheng,[a,b] Liyun Cao,[*a] Gaoxuan Xing,[b] Zongquan Bai,[b] Jianfeng Huang,[a] and Zhiping Zhang[*b]*

[a] *School of Material Science and Engineering, Shaanxi University of Science and Technology, Xi'an 710021, China; Email: caoliyun@sust.edu.cn (L.C.).*

[b] *School of Chemistry and Chemical Engineering, Xi'an Shiyou University, Xi'an 710065, China. Fax: +86 29 8838 2693; Tel: +86 29 8838 2694; E-mail: zhangzp0304@gmail.com (Z.Z.).*

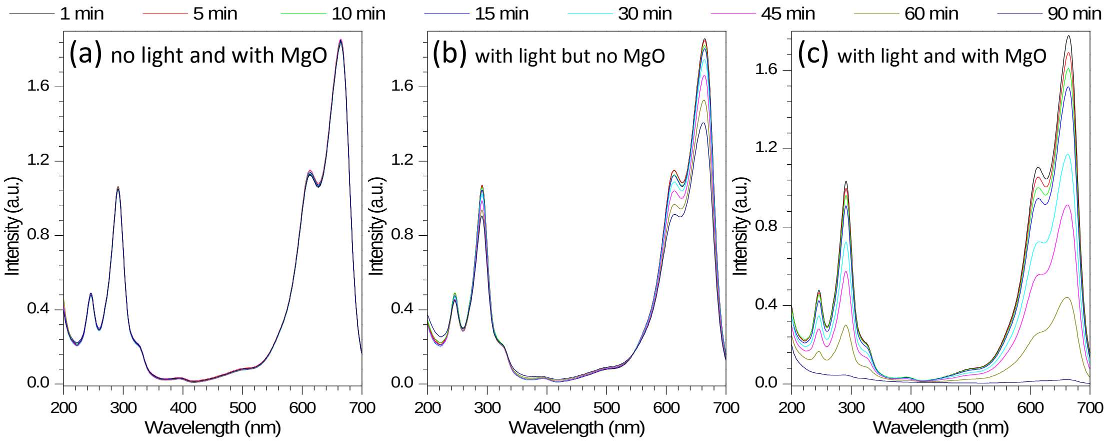

Figure S1. *UV-vis spectra of initial methylene blue solution (100 mg L^{-1}) after treatment under different experimental conditions:* ***(a)*** *no light and with MgO,* ***(b)*** *with light but not MgO, and* ***(c)*** *with light and with MgO.*

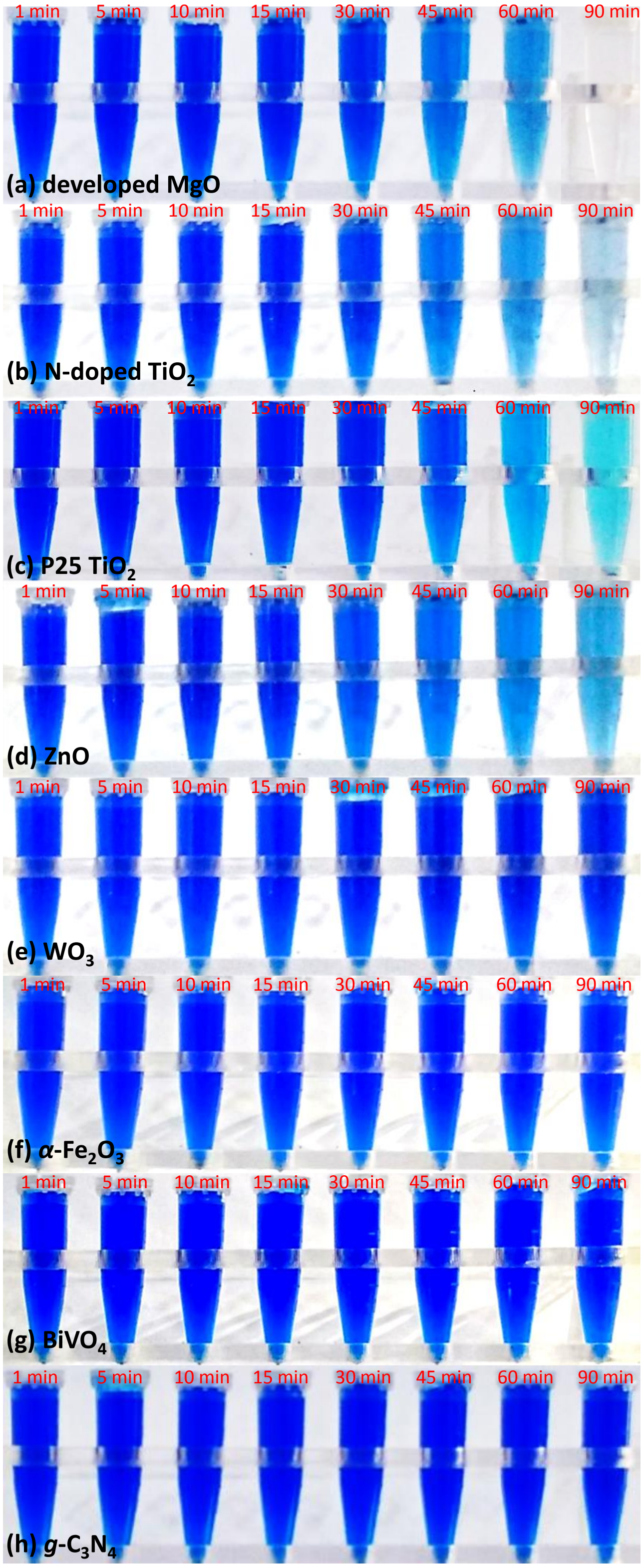

Figure S2. *Photographic images of 100 mg* L^{-1} *methylene blue solution after photocatalysis using* ***(a)*** *developed MgO particles,* ***(b)*** *N-doped* TiO_2*,* ***(c)*** *P25* TiO_2*,* ***(d)*** *ZnO,* ***(e)*** WO_3*,* ***(f)*** *α-*Fe_2O_3*,* ***(g)*** $BiVO_4$*, and* ***(h)*** *g-*C_3N_4 *with variation of irradiation times.*

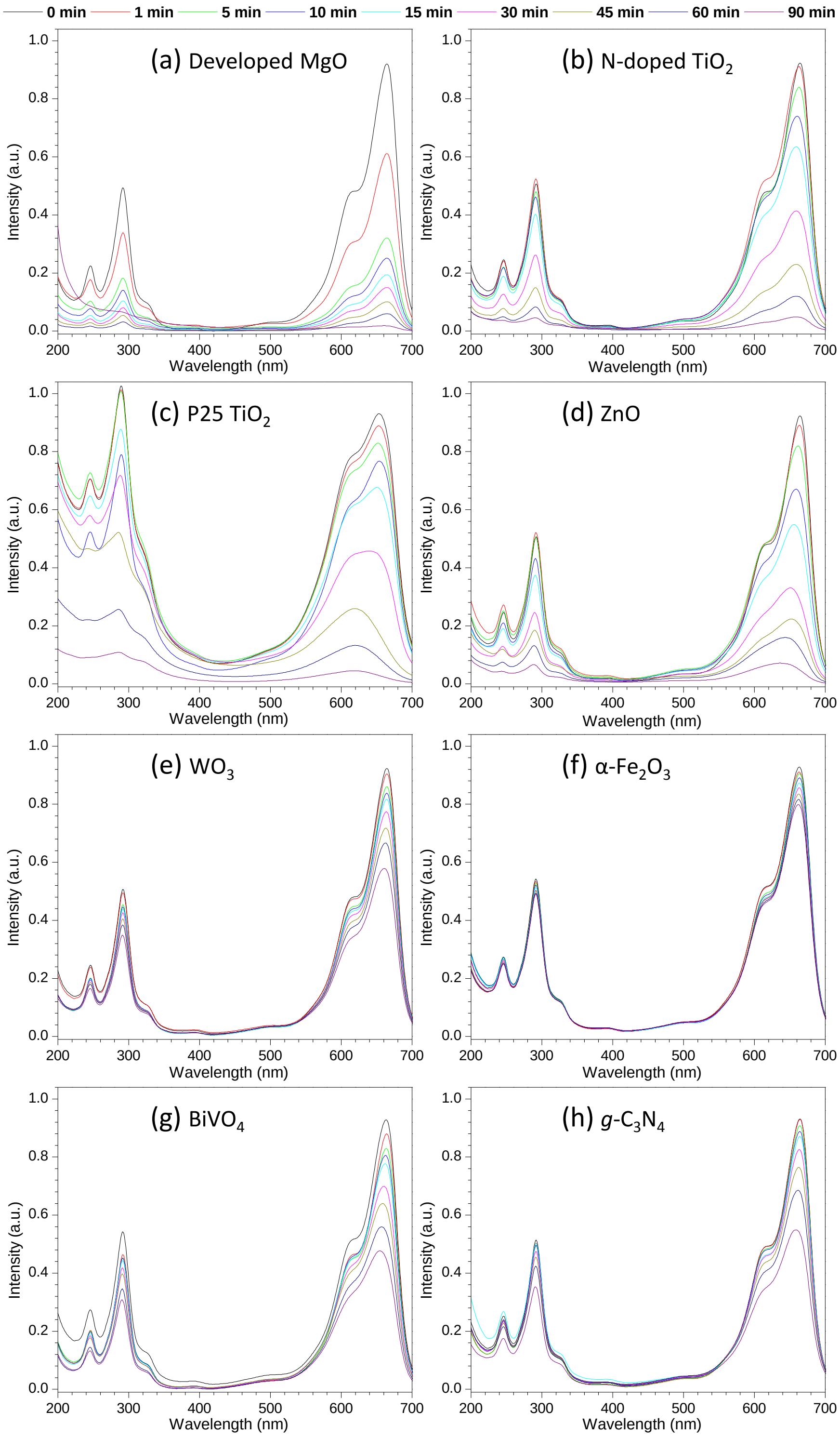

Figure S3. *UV-vis spectra of initial methylene blue solution (100 mg L^{-1}) after treatment using* ***(a)*** *developed MgO particles,* ***(b)*** *N-doped TiO_2,* ***(c)*** *P25 TiO_2,* ***(d)*** *ZnO,* ***(e)*** *WO_3,* ***(f)*** *α-Fe_2O_3,* ***(g)*** *$BiVO_4$, and* ***(h)*** *g-C_3N_4 with variation of irradiation times.*

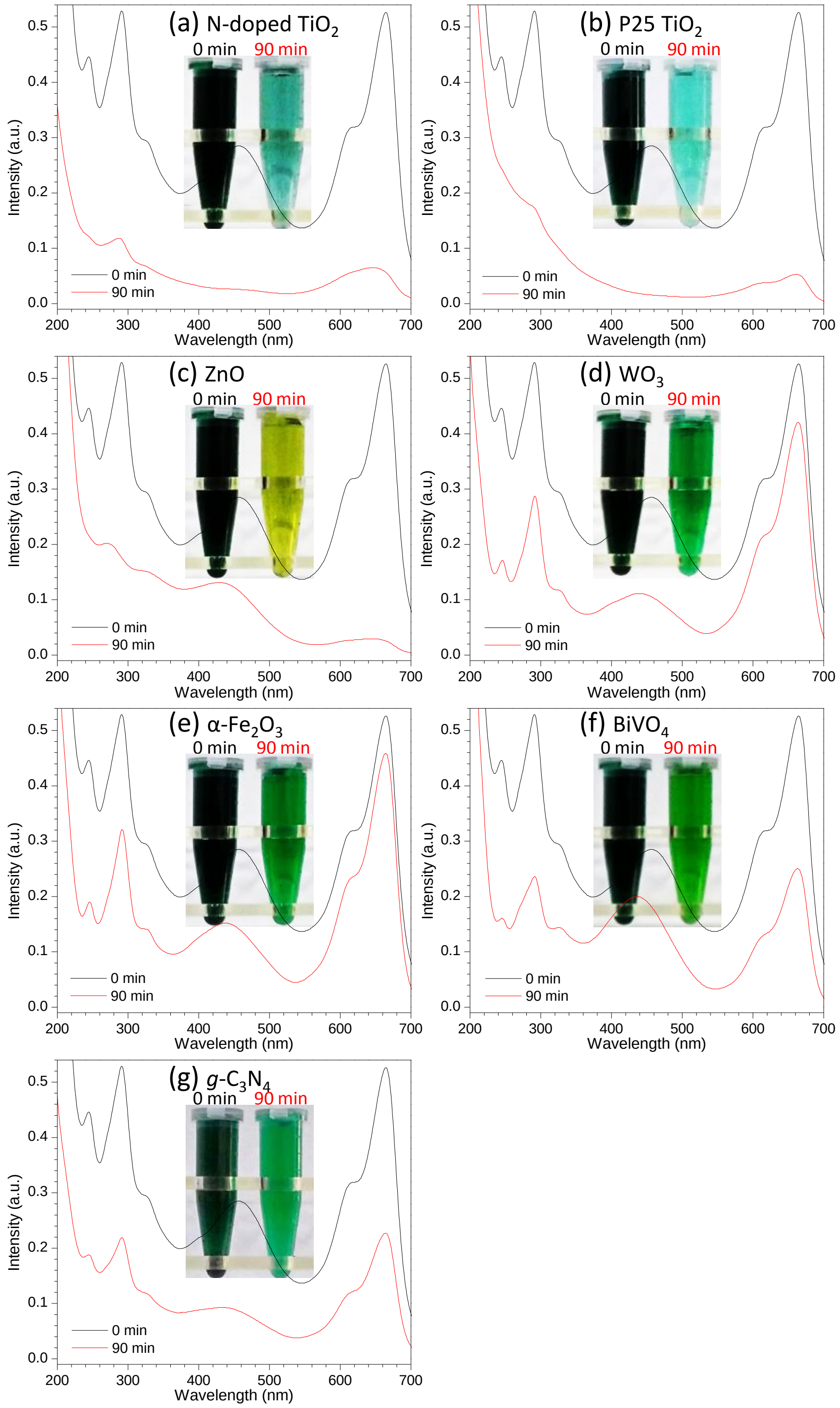

Figure S4. *UV-vis spectra of the mixed organic dyes containing methylene blue, Congo red, thymol blue, bromothmol blue, and eriochrome black T with each concentration of 100 mg* L^{-1} *after treatment using* ***(a)*** *N-doped* TiO_2*,* ***(b)*** *P25* TiO_2*,* ***(c)*** *ZnO,* ***(d)*** WO_3*,* ***(e)*** *α-* Fe_2O_3*,* ***(f)*** $BiVO_4$*, and* ***(g)*** *g-*C_3N_4 *(note: The inset is the photographic image of the corresponding solution after 0 min and 90 min).*

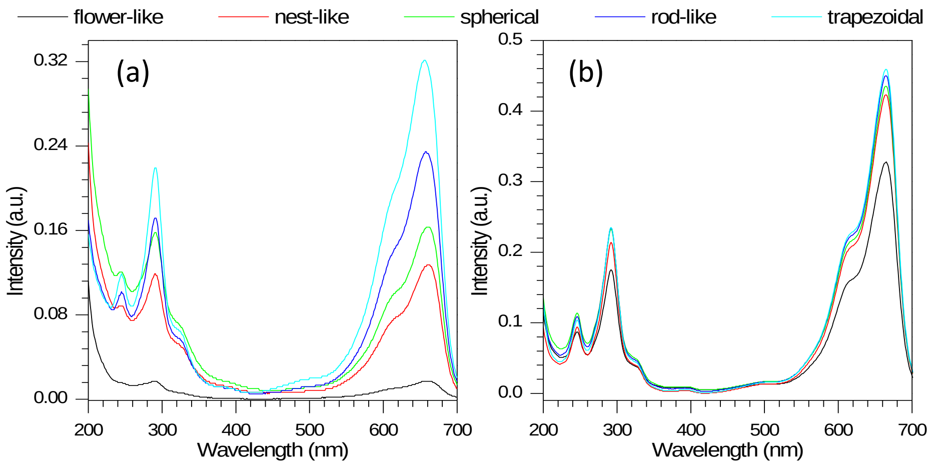

Figure S5. ***(a)*** *UV-vis spectra of initial methylene blue solution (100 mg L^{-1}) after photocatalysis with different morphologies of MgO particles,* ***(b)*** *UV-vis spectra of initial methylene blue solution (50 mg L^{-1}) after adsorption with different morphologies of MgO particles without any UV irradiation.*

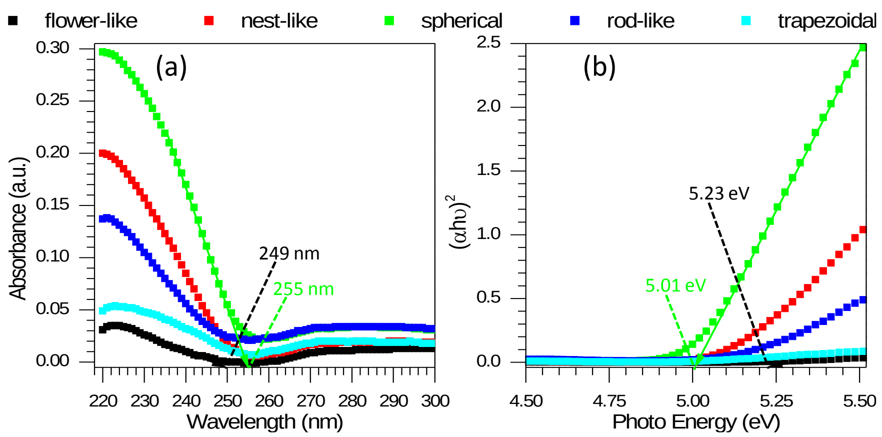

***Figure S6. (a)** UV DRS and **(b)** plot of $(\alpha h\upsilon)^2$ vs $(h\upsilon)$ of different morphologies of MgO particles.*

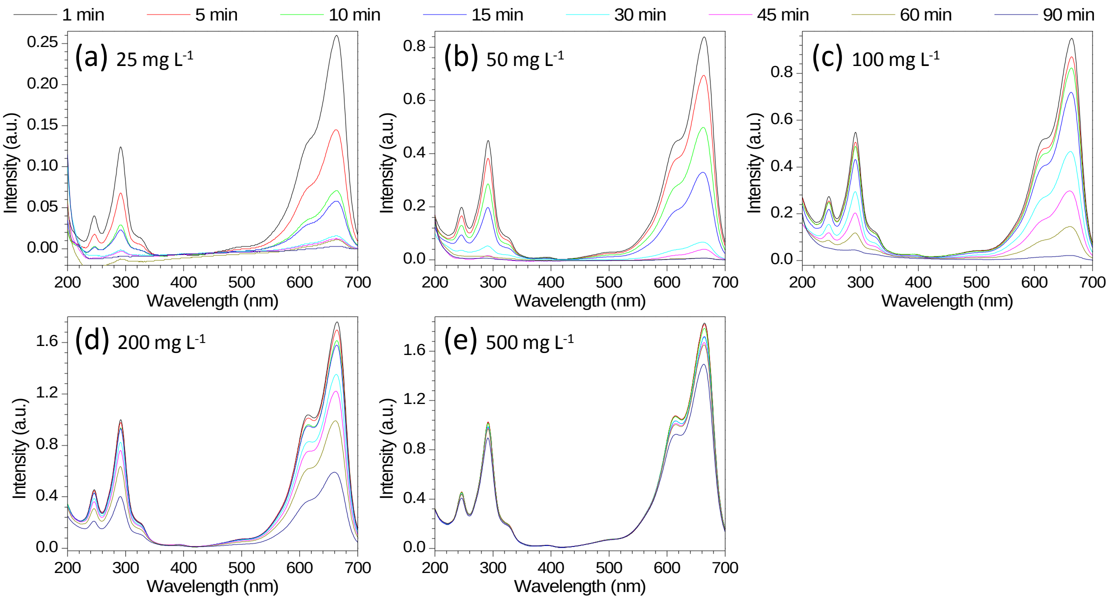

Figure S7. *UV-vis spectra of initial methylene blue solution with different concentrations (25 -500 mg L^{-1}) after photocatalysis using as-prepared flower-like MgO particles under different irradiation times:* ***(a)*** *25 mg L^{-1},* ***(b)*** *50 mg L^{-1},* ***(c)*** *100 mg L^{-1},* ***(d)*** *200 mg L^{-1}, and* ***(e)*** *500 mg L^{-1}.*

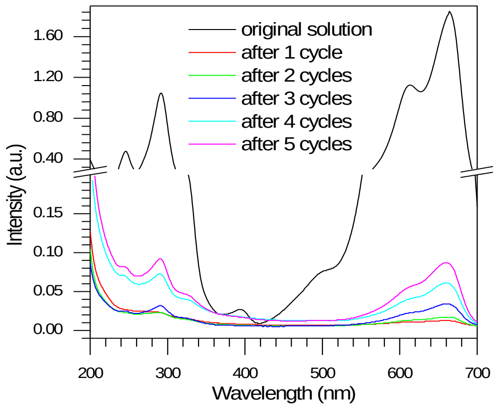

Figure S8. *UV-vis spectra of initial methylene blue solution with a concentration of 100 mg L^{-1} after photocatalysis using as-prepared flower-like MgO particles with different cycles as marked in this figure (irradiation time: 90 min).*

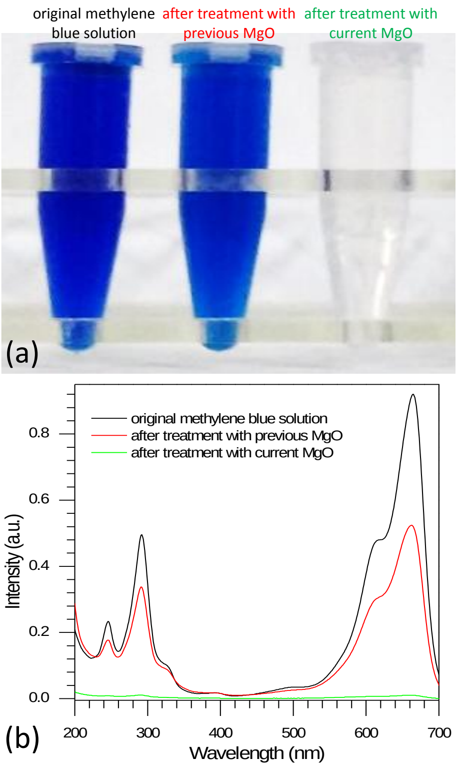

Figure S9. ***(a)****Photographic images and* ***(b)*** *UV-vis spectra of initial methylene blue solution with a concentration of 100 mg* L^{-1} *after photocatalysis using the flower-like MgO in our previous study (CrystEngComm,* ***2018****, 20, 4090-4098) and in the current study (irradiation time: 90 min).*

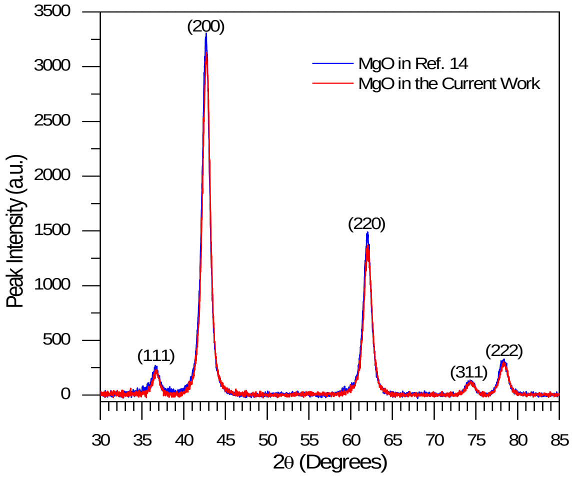

Figure S10. *Comparison of the XRD pattern of the flower-like MgO prepared in the previous report (Reference 14 in the main text and the current study.*
